# Exposure to gold nanoparticles produces cardiac tissue damage that depends on the size and duration of exposure

**DOI:** 10.1186/1476-511X-10-205

**Published:** 2011-11-10

**Authors:** Mohamed Anwar K Abdelhalim

**Affiliations:** 1 Department of Physics and Astronomy, College of Science, King Saud University, Saudi Arabia

**Keywords:** gold nanoparticles, size, heart muscle, histology, inflammatory, nanotoxicity, cytoplasmic vacuolization, rats

## Abstract

**Background:**

Current research focuses on cancer therapy, diagnostics and imaging, although many challenges still need to be solved. However, for the application of gold nanoparticles (GNPs) in therapy and diagnostics it is necessary to know the bioaccumulation and local or systemic toxicity associated to them. The aim of the present study was to investigate the effects of intraperitoneal administration of GNPs on the histological alterations of the heart tissue of rats in an attempt to cover and understand the toxicity and the potential role of GNPs in the therapeutic and diagnostic applications.

**Methods:**

Animals were randomly divided into 3 GNPs-treated rats groups and one control group (CG). The 10, 20 and 50 nm GNPs were administered intraperitonealy at the rate of 3 or 7 days as follows: Group 1: received infusion of 100 μl GNPs of size 10 nm for 3 or 7 days; Group 2: received infusion of 100 μl GNPs of size 20 nm for 3 or 7 days; Group 3: received infusion of 100 μl GNPs of size 50 nm for 3 or 7 days. Control group: received no GNPs.

**Results:**

In comparison with the respective control rats, GNPs-treated rat received 100 μl of 10 and 20 nm particles for 3 days or 7 days demonstrating congested heart muscle with prominent dilated blood vessels, scattered and extravasations of red blood cells, focus of muscle hyalinosis, disturbed muscle fascicles, dense prominent focus of inflammatory cells infiltrate by small lymphocytes and few plasma cells while GNPs-treated rat received 100 μl of 50 nm particles for 3 or 7 days demonstrating benign normal looking heart muscle with normal muscle direction and fascicles, and very few scattered small lymphocytes.

**Conclusions:**

The histological alterations induced by intraperitoneal administration of GNPs were size-dependent with smaller ones induced more affects and related with time exposure of GNPs. This study suggests that interaction of GNPs with proteins and various cell types might be evaluated as part of the toxicological assessment in addition to further experiments related to tissues antioxidant enzymes, oxidative parameters, lipid peroxidation, production of free radicals and/or ROS and cytokine, histomorphologcal and ultrastrucural will be performed to cover and understand the toxicity and the potential use of GNPs as therapeutic and diagnostic tool.

## Introduction

The NPs are being investigated for gene delivery purposes [1-3] and cancer therapy [4]. Data concerning the behavior and toxicity of particles mainly comes from studies on inhaled NPs [5].

NPs may differ in reactivity and solubility and may interact with all kinds of endogenous proteins, lipids, polysaccharides and cells. A series of tests was proposed for evaluation of the toxicity of NPs used in drug delivery systems [6]. GNPs can easily enter cells and the demonstration that amine and thiol groups bind strongly to GNPs has enabled their surface modification with amino acids and proteins for biomedical applications [7-9].

Gold in its bulk form has been considered an inert, noble metal with some therapeutic and medicinal value. GNPs are thought also to be relatively non-cytotoxic [10] while the metallic nature of the metal derived NPs and the presence of transition metals encourages the production of reactive oxygen species (ROS) leading to oxidative stress [9,11,12].

The use of NPs as drug carriers may reduce the toxicity of the incorporated drug [12]. There are differing reports of the extent of the toxic nature of these particles owing to the different modifications of the GNPs, surface functional attachments and shape and diameter size of the NPs [13,14].

The particle size-dependent organ distribution of GNPs has been studied in vivo [15-17]. In vivo studies in rats exposed to aerosols of GNPs revealed that the NPs were rapidly taken into the system with the highest accumulation in the lungs, aorta, esophagus and olfactory bulb [18].

In order to understand and categorize the mechanisms for GNPs toxicity, histological data is needed on the response of living systems to the presence of GNPs of varying size, shape, surface, and exposure duration.

The histological and histochemical characterization of the heart tissues due to GNPs has not been documented and identified before. In the present study, an attempt has been made to characterize the possible histological alterations in the heart tissues after intraperitoneal administration of GNPs and, if so, whether are related to the size of these GNPs and the time of exposure.

## Materials and methods

### Gold nanoparticles

GNPs of different sizes (10, 20 and 50 nm; products MKN-Au-010, MKN-Au-020 and MKN-Au-050, Canada, respectively) were purchased. All GNPs used in this study were in aqueous solution at a concentration of 0.01%. The mean size and morphology of these GNPs were evaluated from transmission electron microscope (TEM) images.

### Animals

A total of 40 healthy male Wistar-Kyoto rats were obtained from the Laboratory Animal Center (College of Pharmacy, King Saud University, Saudi Arabia). The rats nearly of the same age (12 weeks old) and weighing 220-240 g of King Saud University colony were used. Animals were randomly divided into 3 GNPs-treated rats groups and one control group (CG). The 10, 20 and 50 nm GNPs were administered intraperitonealy at the rate of 3 or 7 days as follows: Group 1: received infusion of 100 μl GNPs of size 10 nm for 3 or 7 days (n = 10); Group 2: received infusion of 100 μl GNPs of size 20 nm for 3 or 7 days (n = 10); Group 3: received infusion of 100 μl GNPs of size 50 nm for 3 or 7 days (n = 10). Control group: received no GNPs (n = 10).

The rats were maintained on standard laboratory rodent diet pellets and housed in humidity and temperature-controlled ventilated cages on a 12 h day/night cycle. All experiments were conducted in accordance with the guidelines approved by King Saud University Local Animal Care and Use Committee.

Fresh portions of the heart from each rat were cut rapidly, fixed in neutral buffered formalin (10%), then dehydrated, with grades of ethanol (70, 80, 90, 95 and 100%). Dehydration was then followed by clearing the samples in 2 changes of xylene.

Samples were then impregnated with 2 changes of molten paraffin wax, then embedded and blocked out. Paraffin sections (4-5 um) were stained with hematoxylin and eosin (the conventional histological stain) according to Pearse [19]. The bright-field images were acquired using a Nikon Eclipse 800 microscope equipped with a Nikon DXM1200 color CCD camera (Nikon Instruments Inc., Melville, NY). Stained sections of control and treated rats were examined for histological alterations in the heart tissues.

## Results and discussions

### Size and morphology of gold nanoparticles

The 10 and 20 nm GNPs show spherical shape while 50 nm GNPs show hexagonal shape. The mean size for GNPs was calculated from the images taken by the transmission electron microscope (TEM): The 10 nm GNPs was of mean size 9.45 ± 1.33 nm, 20 nm GNPs was of mean size 20.18 ± 1.80 and the 50 nm GNPs was of mean size 50.73 ± 3.58 [20-23].

### Histological alterations

No mortality occurred for the administration periods 3 and 7 days of GNPs in any of the experimental groups of the present investigation, and no alterations were observed in the appearance and behavior of GNPs treated rats in comparison with the control ones.

**Figure 1:**
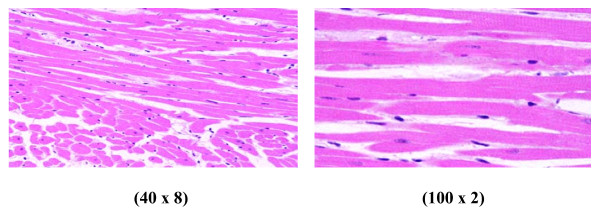
**GNPs-normal rat demonstrating normal heart muscle**.

Control group (Figure 1): Microscopic pictures show GNPs-normal rat demonstrating benign blunt looking heart muscle of different heart muscle directions and with no pathological findings.

**Figure 2:**
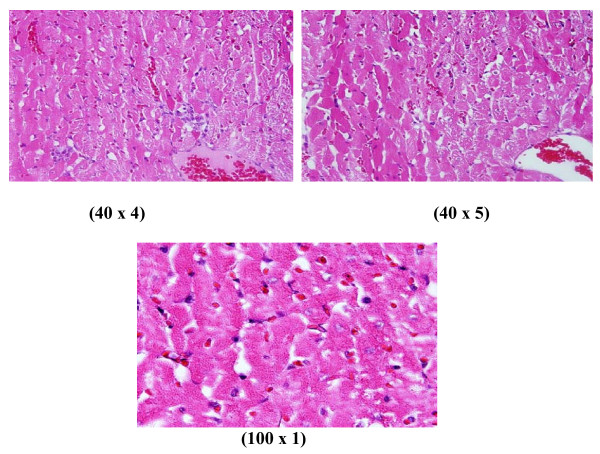
**GNPs-treated rat received 100 μl of 10 nm particles for 3 days demonstrating heart muscle with prominent dilated congested blood vessels, scattered and extravasations of red blood cells and few small lymphocytic infiltrate**.

**Figure 3:**
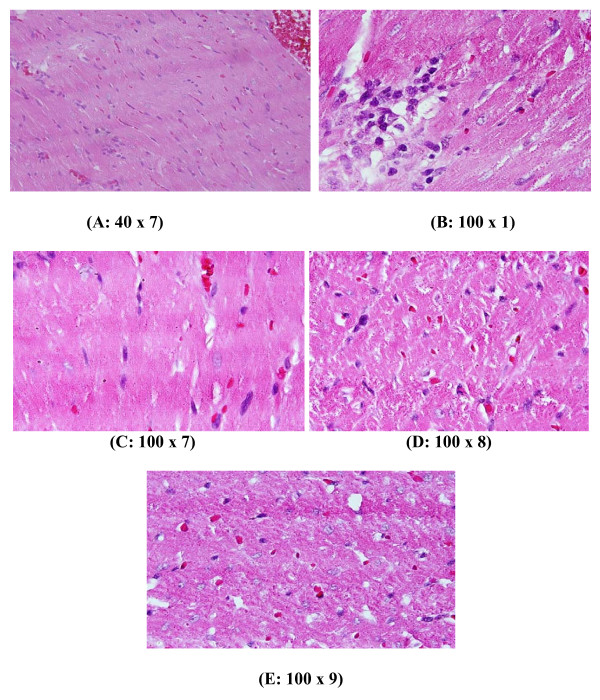
**GNPs-treated rat received 100 μl of 10 nm particles for 7 days demonstrating scattered and extravasations of red blood cells, congested dilated blood vessels, prominent focus of small lymphocytic infiltrate associated with focus of muscle hyalinosis, disturbed muscle fascicles and scattered red blood cells**.

**Figure 4:**
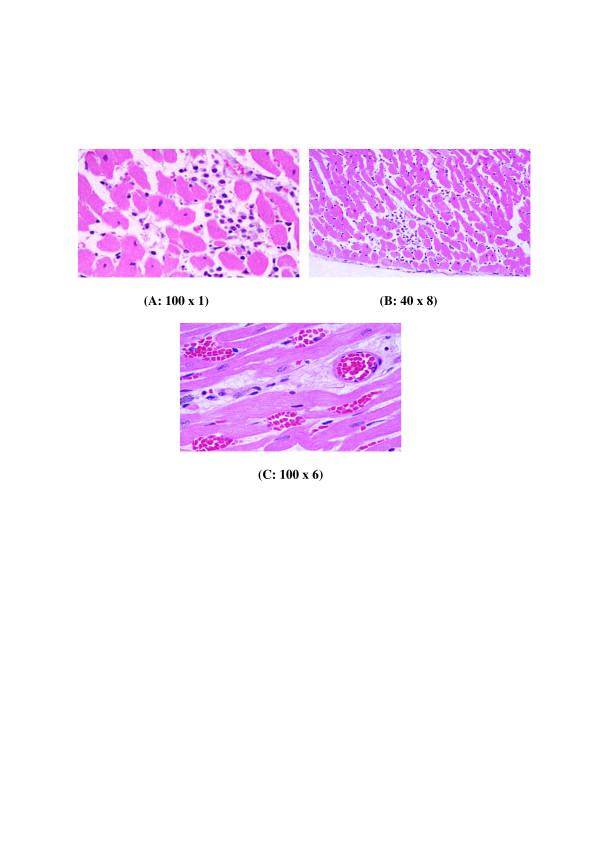
**GNPs-treated rat received 100 μl of 20 nm particles for 3 days demonstrating dense prominent focus of inflammatory cells infiltrate of small lymphocytes and few plasma cells and prominent congested dilated blood vessels and few scattered extravasation red blood cells**.

**Figure 5:**
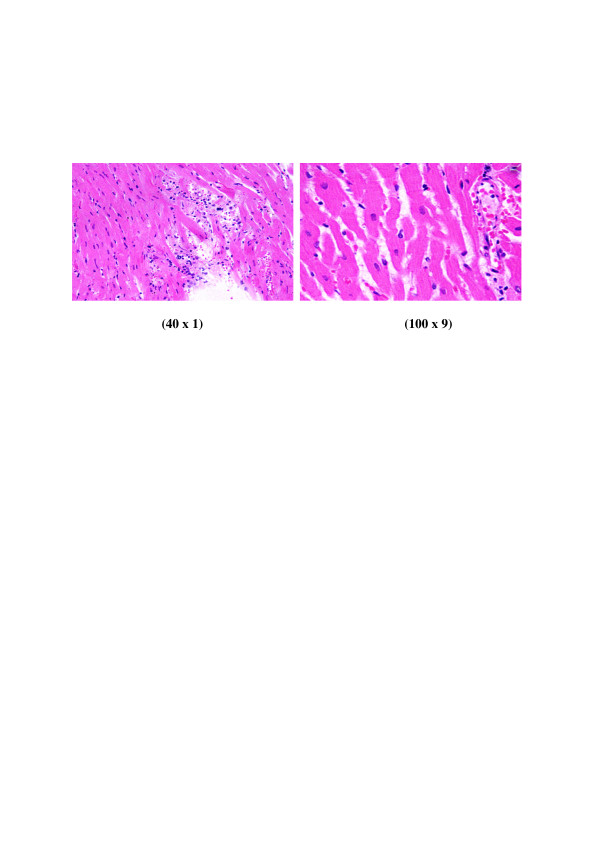
**GNPs-treated rat received 100 μl of 20 nm particles for 7 days demonstrating dense chronic inflammatory cells infiltrate with extravasation of red blood cells**.

**Figure 6:**
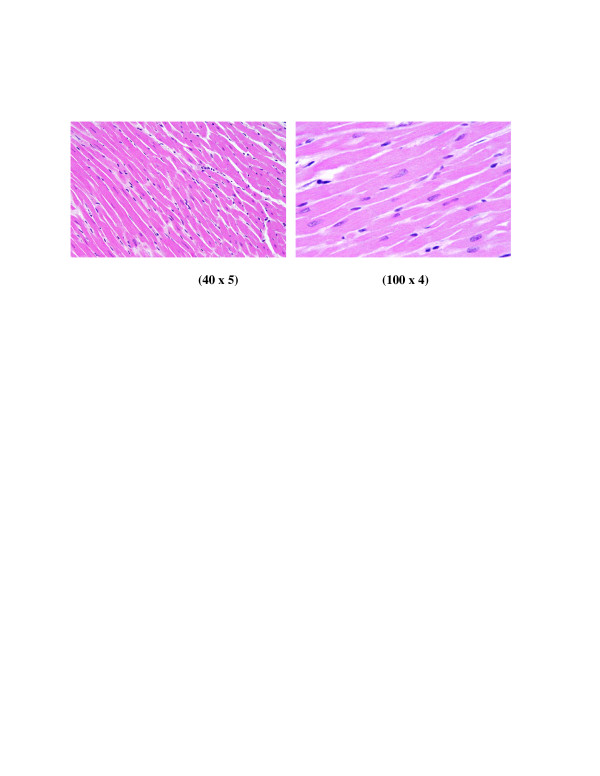
**GNPs-treated rat received 100 μl of 50 nm particles for 3 days demonstrating benign normal looking heart muscle with normal muscle direction and fascicles with no pathological effect**.

**Figure 7:**
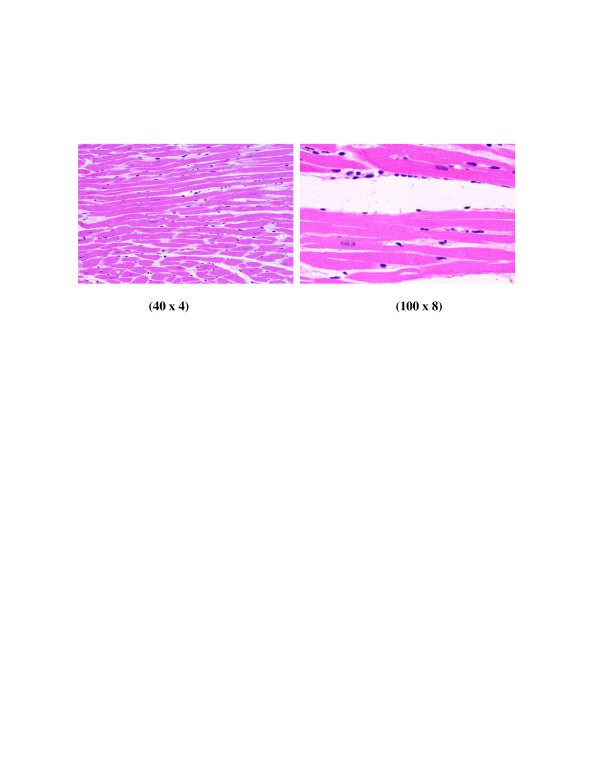
**GNPs-treated rat received 100 μl of 50 nm particles for 7 days demonstrating benign normal looking heart muscle, very few scattered small lymphocytes with no any other pathological effect**.

In comparison with the control group, the following histological alterations were detected in the heart tissue of GNPs-treated rats. These histological alterations were observed in Figures 2, 3, 4, 5, 6, 7 and can be summarized as follows:

**1) **GNPs-treated rat received 100 μl of 10 nm particles for 3 days demonstrating heart muscle with prominent dilated congested blood vessels, scattered and extravasations of red blood cells and few small lymphocytic infiltrate as shown in Figure 2

**2) **GNPs-treated rat received 100 μl of 10 nm particles for 7 days demonstrating scattered and extravasations of red blood cells, congested dilated blood vessels, prominent focus of small lymphocytic infiltrate, focus of muscle hyalinosis, disturbed muscle fascicles as shown in Figure 3.

**3) **GNPs-treated rat received 100 μl of 20 nm particles for 3 days demonstrating dense prominent focus of inflammatory cells infiltrate by small lymphocytes, few plasma cells, prominent congested dilated blood vessels and few scattered extravasations of red blood cells as shown in Figure 4.

**4) **GNPs-treated rat received 100 μl of 20 nm particles for 7 days demonstrating dense chronic inflammatory cells infiltrate with extravasations of red blood cells as shown in Figure 5.

**5) **GNPs-treated rat received 100 μl of 50 nm particles for 3 days demonstrating benign normal looking heart muscle with normal muscle direction and fascicles with no pathological effect as shown in Figure 6

**6) **GNPs-treated rat received 100 μl of 50 nm particles for 7 days demonstrating benign normal looking heart muscle, very few scattered small lymphocytes as shown in Figure 7.

The histological heart alterations induced by intraperitoneal administration of GNPs were size-dependent with smaller ones induced more affects and related with time exposure of GNPs.

This infiltration of GNPs was more prominent after 7 days of administration and in rats received 10 and 20 nm GNPs than those received 50 nm GNPs. The histological heart alterations may suggest that GNPs could interfere with the antioxidant defense mechanism and leading to reactive oxygen species (ROS) generation which in turn may imitate an inflammatory response. Inflammatory cells infiltration was seen in the portal triads and the perioral zones of GNPs treated rats. The infiltrate cells were mainly lymphocytes and plasma cells [20-23].

GNPs were more strongly oxidizing as evidenced by lipid peroxidation [24,25] as well as decreased neutral red retention time (NRRT) and numbers of thiol-containing proteins evident in electrophoretic separations. Cadmium may displace iron or copper from metalloproteins leading to oxidative stress via the Fenton reaction [26].

It has been reported that 5 nm GNPs caused significantly greater oxidative stress and cytotoxicity effects than larger ones [27-29]. The 5 nm GNPs have shown to catalyze nitric oxide (NO) production from endogenous S-nitroso adducts with thiol groups in blood serum. NO reacts rapidly with superoxide producing peroxynitrite (ONOO-) which can interact with lipids, DNA, and proteins via direct oxidative reactions or via indirect radical-mediated damage [28]. ROS production could result from the proportionately high surface area of GNPs used in this investigation [30,31].

Several possible mechanisms of action for the toxicity of particles including injury of epithelial tissue [31], inflammation, and oxidative stress response [32,33]. At the cellular level oxidative stress is considered to be of importance [34,35]. Nanoparticle induced oxidative stress responses in keratinocytes, macrophages and blood monocytes after in vitro exposure [36,37].

NPs are nearly of same dimensions as some biological molecules such as proteins and nucleic acids. Many of these biomolecules consist of long macromolecular chains which are folded and shaped by cooperative and weak interaction between side groups. The GNPs may intrude into these complex folded structures.

GNPs activate the phagocytic activity of the sinusoidal cells by increasing the number of Kupffte cells to help in removing the accumulated GNPs where lysosomes are involved in the intracellular breakdown into small metabolic products. The produced Kupffer cells hyperplasia might be correlated with the amount of injurious to the hepatic tissue induced by GNPs intoxication and represents a defense mechanism of detoxification. Kupffer cell hyperplasia is contributed to hepatic oxidative stress [20-23,38].

This scattered cytoplasmic vacuolization might indicate toxicity effect that exhibited as a result of disturbances of membranes function which leads to massive influx of water and Na^+ ^due to GNPS effects. Cellular swelling might be accompanied by leakage of lysosomal hydrolytic enzymes that lead to cytoplasmic degeneration and macromolecular crowding [39].

Fatty change was observed in some swelling hepatocytes of rats exposed to 100 μl of 10 nm GNPs and to lesser extent in the ones exposed to larger particles. This hepatic liposis was more prominent in rat exposed to GNPs for 7 days than those received the treatment for 3 days [20-23]. Hepatocytes fatty change might be due to lipid peroxidation that leads to rough endoplasmic damage and detachment of the cytoplasmic lipoprotein which indicate abnormal fat metabolism [22,25-27].

The rats received 10 and 20 nm GNPs showed hemorrhage and excess extravasation of red blood cells. Less disruption was observed in rats exposed to 50 nm GNPs while more damage was detected after 7 days than 3 days of GNPs exposure. This alteration might indicate heart muscle damage and congestion by GNPs exposure.

None of the above alterations were observed in the heart muscle of any member of the control group.

The interaction of NPs with living systems is also affected by the characteristic dimensions. As noted above, GNPs, of smaller size, may reach inside biomolecules, a situation not possible for larger GNPs. It has been reported that inhaled NPs reach the blood and may reach other target sites such as the liver, heart or blood cells [40,41].

Reduction in size results in an enormous increase of surface to volume ratio, so relatively more molecules of the chemical are present on the surface, thus enhancing the intrinsic toxicity. This may be one of the reasons that smaller GNPs are generally more toxic than larger particles of the same insoluble material when compared on a mass dose base [42].

In the present study we have not measured GNPs concentration in urine and feces, but this point will be taken into our consideration in other new additional experiments.

Further experiments related to tissues antioxidant enzymes, oxidative parameters, lipid peroxidation, production of free radicals and/or ROS and cytokine, histomorphologcal and ultrastrucural will be performed to cover and understand the toxicity and the potential use of GNPs as therapeutic and diagnostic tool.

## Conclusions

In comparison with the respective control rats, histological alterations induced in the heart tissue exposure could be an indication of congested heart muscle with dilated blood vessels, extravasations of red blood cells, muscle hyalinosis, disturbed muscle fascicles, inflammatory cells infiltrate by small lymphocytes and plasma cells due to GNPs toxicity that became unable to deal with the accumulated residues resulting from metabolic and structural disturbances caused by these particles. One might conclude that these alterations are size-dependent with smaller ones induced more damage with relation to the time exposure of GNPs.

The GNPs-treated rat received 100 μl of 50 nm particles for 3 or 7 days demonstrating benign normal looking heart muscle with normal muscle direction and fascicles, very few scattered small lymphocytes, and with no other pathological effects.

One mechanism of toxicity of NPs is likely to be induction of ROS and the consequential oxidative stress in cells and organs. The appearance of congested heart muscle with prominent dilated blood vessels and focus of inflammatory cells infiltrate by small lymphocytes and plasma cells may suggest that GNPs interact with proteins and enzymes of the hepatic tissue interfering with the antioxidant defense mechanism and leading to reactive oxygen species (ROS) generation which in turn may induce stress.

## Competing interests

The author declares that he has no competing interests.

## Authors' contributions

AMAK has analyzed data, interpreted and written the final draft of this manuscript. The animal model used in this study was obtained from the Laboratory Animal Center (College of Pharmacy, King Saud University, Saudi Arabia). AMAK has conceived the study and its design and obtained research grants for this study. The authors have read and approved the final manuscript.
